# Validation of the global resource of eczema trials (GREAT database)

**DOI:** 10.1186/s12895-015-0024-z

**Published:** 2015-03-14

**Authors:** Helen Nankervis, Alison Devine, Hywel C Williams, John R Ingram, Elizabeth Doney, Finola Delamere, Sherie Smith, Kim S Thomas

**Affiliations:** Centre of Evidence Based Dermatology, School of Medicine, University of Nottingham, Nottingham, UK; Glan Clwyd Hospital, Betsi Cadwaladr University Health Board, Bodelwyddan, Rhyl UK; Department of Dermatology & Wound Healing, Institute of Infection & Immunity, Cardiff University, Cardiff, Wales; Cochrane Skin Group, Centre of Evidence Based Dermatology, University of Nottingham, Nottingham, UK

**Keywords:** Validation, Database, Randomised controlled trials, Eczema, Atopic dermatitis

## Abstract

**Background:**

Eczema (syn. Atopic Eczema or Atopic Dermatitis) is a chronic, relapsing, itchy skin condition which probably results from a combination of genetic and environmental factors. The Global Resource of EczemA Trials (GREAT) is a collection of records of randomised controlled trials (RCTs) for eczema treatment produced from a highly sensitive search of six reference databases. We sought to assess the sensitivity of the GREAT database as a tool to save future researchers repeating extensive bibliographic searches.

**Methods:**

All Cochrane systematic review on treatments for eczema and five non-Cochrane systematic reviews on eczema were identified as a reference set to assess the utility of the GREAT database in identifying randomised controlled trials (RCTs). RCTs included in the systematic reviews were checked for inclusion in the GREAT database by two independent authors. A third author resolved any disagreements.

**Results:**

Five Cochrane and six non-Cochrane systematic reviews containing a total of 105 RCTs of eczema treatments were included. Of these, 95 fitted the inclusion criteria for the GREAT database and 88 were published from 2000 onwards. Of the 88 eligible studies, 92% were found in the GREAT database. Seven trials were not included in the GREAT database - two of these were reported within a review paper and one as an abstract with no trial results.

**Conclusions:**

The sensitivity of the GREAT database for trials from 2000 onwards was high (75/88 trials, 94%). Sensitivity for the period prior to 2000 was less sensitive, due to differences in how the trials were identified prior to this time.

‘Dual’ filtering for new records has recently become part of the GREAT database methodology and should further improve the sensitivity of the database in time. The GREAT database can be considered as a primary source for future systematic reviews including randomised controlled trials of eczema treatments, but searches should be supplemented by checking reference lists for eligible trials, searching trial registries and contacting pharmaceutical companies for unpublished studies.

## Background

Eczema is a chronic, relapsing, itchy skin condition (syn. Atopic Eczema or Atopic Dermatitis) which is the result of a combination of genetic and environmental factors [1].

Searching for published reports of randomised controlled trials (RCTs) can be time consuming and complex. In order to facilitate the speedy identification of RCTs of eczema treatments, the Global Resource of EczemA Trials database (GREAT) was created. This free, publicly accessible database (www.greatdatabase.org.uk) contains summary details of published randomised controlled trials (RCTs) on the treatment of eczema. Trials are identified using a highly sensitive search of six databases [2]. The aim of the GREAT database is to save research time by: 1) creating only one database to search for trials of eczema treatment; and 2) providing enough pertinent information about each trial to allow systematic reviewers and guideline writers to decide whether the trial fits their inclusion criteria without having to obtain and scrutinise the full papers for all possible trials themselves.

If potential users of the GREAT database are not convinced that it will provide a comprehensive list of all eczema RCTs, then they are far less likely to use it as their main database to search and will conduct their own searches. This will not save research time or help to prevent research wastage as was the original intention.

The objective of this study was to evaluate the sensitivity of the GREAT database by comparing those studies found in systematic reviews with those in the GREAT database. Sensitivity was defined as the number of relevant RCTs identified in the GREAT database, divided by the total number of relevant RCTs in the included systematic reviews.

## Methods

All methods described below were undertaken by two authors independently, with any discrepancies being resolved by another author acting as an arbiter.

As there is no obvious ‘gold standard’ resource of trials of eczema treatments, systematic reviews were considered the ‘gold standard’ and the GREAT database was the ‘comparator’.

It was decided, a priori, to restrict the comparison of the GREAT database to all published Cochrane reviews of eczema treatments, and five non-Cochrane reviews of eczema treatments. The latter were those with the most recent search end dates. Reviews also had to have a full list of citations for the included studies and sufficient details of the review’s inclusion criteria to allow a fair comparison with the GREAT database. The validation only compared included RCTs, as the GREAT database only includes RCTs.

Results of this study are presented as being for the period from 2000 onwards and pre-2000 due to differences in the way that the searches were conducted during these periods. RCTs published prior to 2000 were identified from a previously published systematic review of eczema treatments [3], whereas RCTs published from 2000 onwards were identified prospectively as the GREAT database was developed.

### Identifying systematic reviews of eczema treatments

The mapping of systematic reviews of atopic eczema project developed by the Centre of Evidence Based Dermatology [4,5] has records of systematic reviews from 2000 onwards (including both Cochrane and non-Cochrane reviews). This map of systematic reviews was mined independently by two authors for all Cochrane systematic reviews and five non-Cochrane reviews involving eczema treatments. Any disagreements about which systematic reviews to include as per the pre-specified inclusion criteria were resolved by the arbiter before the list was finalised.

### Identifying included and excluded trials from systematic reviews

Two authors independently scrutinised the included systematic reviews to obtain the list of citations of included RCTs studies (other included studies depending on the nature of the review). Any disagreements between the two authors about the included and excluded studies were resolved by the arbiter. Excluded studies were defined as those described as such in the review text or a table(s). Any trials not in English and not in GREAT had the appropriate data extracted by a researcher with the required language skills, if further information was required.

### Identifying trials from the GREAT database

Two authors independently attempted to find all the included and excluded trials identified from the systematic reviews, using the search facility and exploration of the GREAT database via the browse and filter facilities. Where an included or excluded trial was not found in the GREAT database, the reasons for this were explored and recorded.

The reported inclusion criteria for each systematic review were used to identify any additional RCTs in the GREAT database which could have been eligible for inclusion in the review, but were not in the list of included studies of that review. Where an RCT was identified in the GREAT database that was not in the included studies list of one or more of the systematic reviews, the reasons for this were explored, consulting the full trial paper and GREAT if necessary. Any disagreements between the two author’s results were resolved by the independent arbiter before the results were analysed.

## Results

### Summary of results

A search of the mapping of eczema systematic reviews [4] revealed 59 eczema treatment systematic reviews published since 2000. Of these, six were Cochrane reviews [6-11] and were included in this validation study. Five non-Cochrane systematic reviews [12-16] with the most recent search end dates and which matched all other inclusion criteria were included in the study.

The six Cochrane reviews included 72 randomised controlled trials (RCTs) on eczema treatments and no other types of studies. All of the reviews searched The Cochrane Library, MEDLINE, and Embase; three searched the Allied and Complementary Medicine Database (AMED) [7,9,11]; one searched the Cumulative Index to Nursing and Allied Health Literature (CINAHL) [11]; two searched the Latin American and Caribbean Health Sciences database (LILACS) [7,9]; three searched PsycINFO® [7,9,10]; and two searched the ISI Web of Science [7,9]. Four [6,8,10,11] of the six Cochrane reviews searched various other databases and repositories such as trials databases, the Food and Drug Administration (FDA) website, European Medicines Agency (EMEA) website, and Chinese databases. The Cochrane reviews included in this study covered topical calcineurin inhibitors, antibacterials, dietary interventions, Chinese herbal medicine, psychological and educational interventions and probiotics.

The five non- Cochrane reviews included 33 RCTs on eczema treatments that could be included in this validation study. In addition, 52 other studies included in the non-Cochrane reviews were excluded from the validation study as they were either controlled clinical trials, cohort studies, or RCTs involving other dermatoses.

Four reviews searched the Cochrane Library [12-14,16],, four searched MEDLINE [12,14,15], three searched Embase [12-14]; one searched Current Contents [15], one searched HomInform [15], and two searched PubMed [13,15]. Four [12,14-16] out of five of the reviews undertook manual searching of the reference lists of included trials, reviews and other sources such as textbooks and guidelines. The non-Cochrane reviews included in this study covered homeopathy, azathioprine, topical calcineurin inhibitors, topical corticosteroids.

### Assessment of trials published from 2000 onwards in the GREAT database

#### Trials missing from GREAT but found in systematic reviews

The GREAT database did not contain all the included RCTs for four [6,8,9,14] of the eleven included systematic reviews. There were 13 out of 88 trials in total (see *Table*1) that were not found in the GREAT database. The reasons for this are discussed below.

**Table 1:** **Summary of results for trials from 2000 onwards**

**Review**	**Number of RCTs included in the review**	**Proportion of included eczema RCTs found in GREAT**	**Reasons for RCTs missing from GREAT**	**Sensitivity of the GREAT database (all missing RCTs)**	**Sensitivity of GREAT (RCTs missing in error)**
**Cochrane reviews**					
Ashcroft et al. 2007 [6]	31	23/31	Unpublished trial records obtained from a pharmaceutical company (n = 5) A report of the same trial was present in GREAT, but the citation for the included study was not (n = 2). One trial not included in GREAT in error (n = 1).	(23/31) 74%	(28/31) 87%
Bath-Hextall et al. 2008 [7]	2	2/2	No trials missing from the GREAT database	100%	100%
Birnie et al. 2008 [8]	7	6/7	Trial was not aiming to ‘treat’ the eczema, only reduce bacterial numbers (n = 1).	(6/7) 91%	100%
Boyle et al. 2008 [9]	11	10/11	Filtered incorrectly in error, should have been an included trial (n = 1)	(10/11) 91%	(10/11) 91%
Ersser et al. 2007 [9]	4	4/4	No trials missing from the GREAT database	100%	100%
Zhang et al. 2005 [11]	1	1/1	No trials missing from the GREAT database	100%	100%
**Totals for Cochrane reviews**	**56 included RCTs**	**46/56 RCTs included in the GREAT database**	**10 trials missing from the GREAT database**	**(46/56) 82%**	**(52/56) 93%**
**Non Cochrane Reviews**					
Schram et al. 2011 [12]	2	2/2	No trials missing from the GREAT database	100%	100%
Yin et al. 2011 [13]	4	4/4	No trials missing from the GREAT database	100%	100%
Svensson et al. 2007 [14]	17	14/17	Abstract which did not present information about results (n = 1). Two Japanese phase II RCTs which fit GREAT inclusion criteria reported only within a review, omitted from GREAT in error.(n = 2)	(14/17) 82%	(14/17) 82%
Simonart et al. 2011 [15]	1	1/1	No trials missing from the GREAT database	100%	100%
Schmitt et al. 2011 [16]	8	8/8	No trials missing from the GREAT database	100%	100%
**Totals for non-Cochrane Reviews**	**32 included RCTs**	**29/ 32 RCTs included in the GREAT database**	**3/32 trials missing from the GREAT database**	**(29/32) 91%**	**(29/32) 91%**
**Totals for Cochrane and Non-Cochrane reviews**	**88 included RCTs**	**75/88 RCTs included in the GREAT database**	**13/88 trials missing from the GREAT database**	**(13/88) 85%**	**(81/88) 92%**

Footnote: Totals in bold.

#### Trial missing with reason

One of the trials [17], was included in a Cochrane review but did not fulfil the GREAT inclusion criteria as it assessed changes in bacterial numbers rather than clinical outcomes.

Five trials [18-22] were included in a Cochrane review, but had been identified through a pharmaceutical company trials database [23] and did not contain enough information in the trial records provided to be certain whether these trials appear in the GREAT database or not (see *Table*2).

**Table 2:** **Summary of the sensitivity of the GREAT database**

**Proportion of trials missing from the GREAT database**	**13/88 (15%)**
**Proportion of trials missing in error from the GREAT database (two of these were included, but under a different citation)**	7/88 (8%)
Proportion of trials truly missing from the GREAT database in error (two of these were only cited in a review paper, one as abstract only with no results provided)*	5/88 (6%)

*These five citations have now been added to the GREAT database.

#### Trials missing in error

Seven trials 7/88 (8%) were identified from systematic reviews and fitted the GREAT inclusion criteria, but were not present in the GREAT database (see *Table*2). Two of these trials [24,25], were additional citations for a trial that was already included in the GREAT database. Of the remaining five trials two were reported only within a review paper [26-28] and one [29] was published as a conference abstract and did not report trial results.

### Potential implications of trials missing from GREAT

One Cochrane review from which one of the missing in error trials was included may possibly not have drawn such strong conclusions about the level of problems with adverse event if the trial had not been included. The other two Cochrane reviews and two non-Cochrane reviews from which one of the missing in error trials were included would have been very unlikely to have drawn different conclusions if the trials had not been included.

### Trials published from 2000 onwards found in the GREAT database that potentially should have been included in systematic reviews

For one of the included Cochrane reviews on anti-staphylococcal interventions [8], three trials [30-32] were identified using the GREAT database that were not included in the review. We believe from the trial reports and the reported inclusion criteria of the review, that these three trials should have been included in this review. It is possible that the review authors managed to gather additional information about these trials which resulted in them being excluded; however, as the trials are not listed as excluded studies when they would have been so close to inclusion, this is less likely.

### Assessment of pre 2000 trials missing from the GREAT database

As trials published before 2000 were added to GREAT directly from a systematic review [3], a strict validation comparison was not performed for trials before 2000.

Four trials [33-36] published before 2000 and included in one Cochrane review [8], were not present in the GREAT database. All four trials were returned in the search strategy for the Cochrane systematic review [8] and so they were not found exclusively through hand searching.

It is most likely that the four trials [33-36] were not selected for inclusion in the overarching systematic review that provided the source for pre-2000 RCTs due to errors in the filtering process; or possibly the trials were excluded as they did not match the systematic review [3] inclusion criteria.

## Discussion

The GREAT database is free to use and has been specifically designed to be quick and easy to access by all potential users, including guideline writers, patient information developers, methodologists, healthcare practitioners, and patients. The GREAT database offers much more than a standard bibliographic database; which provides citations and abstracts of articles only. Researchers identifying references from other databases would be well advised to cross-check them in the GREAT database, in order to take advantage of the additional data extraction and analysis available there.

In this validation study, the GREAT database was found to include 92% of the RCTs identified in published systematic reviews, and was also able to identify an additional three trials that had not been included in published reviews.

Two trial reports were counted as missing where the trial itself was reported in the GREAT database, but not the particular citations used in the systematic review. This is something that could helpfully be addressed in the future by linking all reports and abstracts from the same trial to the same trial record in the GREAT database. This would prevent bias being introduced by multiple reporting of the same trial.

Although the GREAT database is comprehensive, if conducting a comprehensive systematic review, other methods of identifying trials may be required, such as searching trials databases, as well as hand-searching and contacting of trial authors. In addition, reviews including studies other than RCTs will require additional searches of bibliographic databases to search for observational and non-randomised studies.

## Conclusions

### Strengths and weaknesses

Although using published systematic reviews as the gold standard for validation of the GREAT database is a useful tool for establishing coverage of the database, direct comparison can be challenging due to differences in eligibility criteria for reviews compared to the GREAT database. This can lead to an apparent lowering of the sensitivity of the database if different eligibility criteria have been applied.

Many treatments that the GREAT database covers have not been assessed in this validation study, as only a selection of systematic reviews were used. Sensitivity of the database may differ depending on the treatment being reviewed and so ongoing validation of the GREAT database is required.

Reviewing the GREAT database against all of the Cochrane systematic reviews of eczema treatments has ensured a complete comparison has been made using a very rigorous search methodology. The main weakness with this comparison is that authors of Cochrane reviews are expected to contact trial authors for information not present in the trial report. The GREAT database methodology does not include this due to time and resource constraints.

One review included RCTs that were identified from a trials database in which most trials were reported to be unpublished. Unfortunately it is impossible to be sure if five of the missing trials are in the GREAT database or not, as insufficient information is available in the public domain. For all eczema trials to be included in GREAT they must be published. Efforts to get all clinical trials registered and published, such as the AllTrials campaign [37], are helping to achieve this.

### Implications for the future development of the GREAT database

One area for improvement of the GREAT database is dual filtering of references in order to reduce errors of omission of potentially eligible RCTs. Dual filtering involves two authors independently assessing the search results to determine eligibility of the included studies, with disagreements being resolved by a third party as necessary. Dual filtering has recently become part of the GREAT database methodology and should further improve the sensitivity of the database. It is our intention to apply dual-filtering retrospectively in order to ensure complete coverage of the GREAT database, but in the meantime, additional searches of bibliographic databases may be required, especially for RCTs published prior to 2000.

Other possible sources for trials to add to the GREAT database could be the interrogation of unpublished trials databases, searching of additional specialist databases such as PsycINFO® or Chinese databases, or adding all citations found for a particular trial (including conference abstracts). Whether or not to widen the inclusion criteria of the GREAT database beyond its current scope requires careful consideration as to its feasibility, impact and resource implications.

The GREAT database is sufficiently sensitive for most users, and is a resource that is quick and simple to interrogate; particularly for those looking for the best evidence for specific treatments that have not yet been included in a full systematic review, or for people looking for new trial evidence published after a systematic review’s search end date. The more the GREAT database is used and added to on an iterative basis, the better it will be.

Although there are other databases, such as for transfusion [38], as far as the authors are aware there are no other databases similar to GREAT for any other dermatological condition. It is therefore particularly important that the impact of the GREAT database on eczema research is assessed in order to enhance its potential to improve patient care and methodology of clinical trials in dermatology research. Over the next 5–10 years, similar databases for other common skin diseases such as psoriasis, acne, hand eczema and skin cancer might be developed with the aim of facilitating more effective mobilisation of evidence for clinical and research uses. The resources required to develop and maintain databases such as GREAT for the public good should not be underestimated. Collating the evidence and designing and testing the database required 20 months of researcher time, specialist IT input and hosting costs. Ongoing updates now require around 2 weeks of researcher time per month.
